# Regulatory mechanisms and applications of *Lactobacillus* biofilms in the food industry

**DOI:** 10.3389/fmicb.2024.1465373

**Published:** 2025-01-07

**Authors:** Peilin Yao, Effarizah Mohd Esah, Chuanping Zhao

**Affiliations:** ^1^Food Technology Division, School of Industrial Technology, Universiti Sains Malaysia, Penang, Malaysia; ^2^School of Biotechnology and Food Engineering, Suzhou University, Suzhou, China

**Keywords:** *Lactobacillus*, biofilm, quorum sensing, stress resistance, encapsulation

## Abstract

*Lactobacillus* is widely recognized for its probiotic benefits and has been widely used in food production. While biofilms are typically associated with pathogenic bacteria, they also served as a self-protective mechanism formed by microorganisms in an adverse environments. In recent years, relevant studies have revealed the excellent characteristics of *Lactobacillus* biofilms, offering new insights into their potential applications in the food industry. The *Lactobacillus* biofilms is important in improving fermentation processes and enhancing the resilience of *Lactobacillus* in various conditions. This paper reviews how quorum sensing regulates the formation of *Lactobacillus* biofilms and explores their roles in stress resistance, bacteriostasis and food production. Additionally, it highlights the emerging concept of fourth-generation probiotics, developed through biofilm technology, as a novel approach to probiotic applications.

## 1 Introduction

In 2002, the joint FAO/WTO Expert Committee introduced a scientifically grounded definition of probiotics, describing them as living microorganisms that confer health benefits upon the host when ingested in adequate quantities (FAO/WHO, 2002). The most widely used probiotic strains belong to *Lactobacillus* spp., *Bifidobacterium* spp., *Lactococcus* spp., *Leuconostoc* spp., *Streptococcus* spp., and various species, with *Lactobacillus* spp being the most extensively studied. As a representative of probiotics, some *Lactobacillus* have good probiotic properties, including lowering intestinal pH, regulating intestinal flora, preventing lactose intolerance, and inhibiting the growth of harmful microbes in the dairy products (Garnier et al., 2020), meat products (Danielski et al., 2020), aquatic products (Aymerich et al., 2019), and fruit and vegetable products (Ma et al., 2019), among others. Furthermore, these bacteria play an important role in the fermentation processes of common foods, such as yogurt and kimchi (Mathur et al., 2020; Wang et al., 2021). To demonstrate efficient biological activities, a daily dose of probiotics of about 10^8^–10^9^ colony-forming units (CFU) is required during their passage through the gastrointestinal tract (Liu et al., 2019). Over time, methods to improve the stress resistance have evolved. These approaches include the use of improved strain protective agent (Sánchez et al., 2017), the addition of oligosaccharides (Dong et al., 2023), gene recombination technology (Wang et al., 2013a) and the encapsulation of probiotics in biofilms, referred to as fourth-generation probiotics (Salas-Jara et al., 2016). Both domestically and internationally, especially within the fermentation industry (Rosche et al., 2009), bioreactors-based biofilms are widely used in sewage treatment (Bao and Dai, 2013), biological fermentation (Cheng et al., 2011), and microbial fuel cells (Raganati et al., 2016).

Probiotics can form complex communities known as biofilms, which offer several advantages for microbial populations when facing various abiotic or biotic stress factors (Salas-Jara et al., 2016). The formation of biofilms allows bacteria to preferentially adhere to specific epithelium surfaces, such as the intestinal mucosa, prolonging and stabilizing their presence in the epithelium and helping to exclude pathogenic bacteria by competitive inhibition or steric hindrance (Saxelin et al., 2005). A key characteristic of biofilms is the formation of extracellular polymers, which provide mechanical stability and promote the formation of a microenvironment, which triggers quorum sensing and further regulates the maturation of the biofilm (Allison, 2003). A mature biofilm has stronger antibacterial activity and stress resistance (Lewis, 2001).

The formation of *Lactobacillus* biofilms is beneficial to the environmental adaptability and probiotic properties of *Lactobacillus*. Therefore, understanding the regulatory mechanisms of *Lactobacillus* biofilm formation and the biofilm’s characteristics contributes to the potential role of *Lactobacillus* on food fermentation industry and promoting human health. However, the relevant knowledge on this topic remains fragmented, highlighting the need for a review on the *Lactobacillus* biofilm and their practical applications.

## 2 Regulatory mechanisms of *Lactobacillus* biofilm formation

**FIGURE 1:**
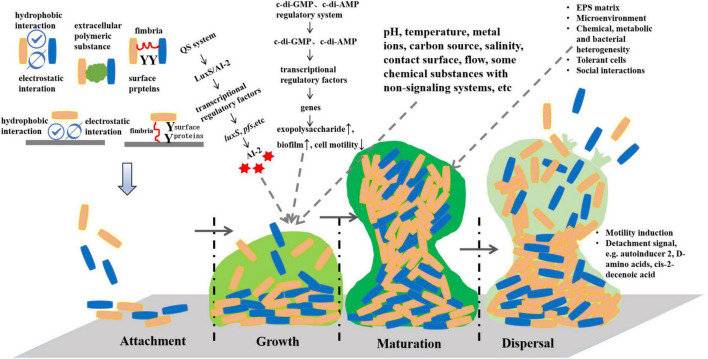
Formation and regulation of lactic acid bacteria biofilms.

The formation of bacterial biofilms is dependent on the regulation of signaling systems, including quorum sensing (QS), second messenger signaling system and cyclic adenosine phosphate-receptor protein (cAMP-CRP) signaling system (Liu et al., 2020; Li and Tian, 2012) (Figure 1). In the existing reports, the QS system is mainly involved in the formation and regulation of *Lactobacillus* biofilm. QS, also known as “autoinduction,” refers to the induction phenomenon in which bacteria employ self-inducing molecules to communicate and coordinate their group behavior (Papenfort and Bassler, 2016). As bacteria grow, they secrete a signal molecule sensing the surrounding environment. By detecting variations in the concentration of these signal molecules, bacteria can modulate the expression of related genes, thereby regulating associated behaviors. Consequently, interfering in bacterial QS signaling to either promote or inhibit the development of lactic acid bacteria (LAB) biofilms holds substantial significance in terms of enhancing intestinal immunity, promoting health, and elevating the quality of fermented food.

### 2.1 AI-2 mediated QS system

The information communication mode of LAB is carried out in a density-dependent manner, which can be divided into intraspecific and interspecific information communication. Intraspecific communication used autoinducing peptides (AIPs) as signal molecules. The precursor AIPs are transcribed and modified to form specific AIPs, which cannot pass through the cell wall freely and must reach the extracellular via the ABC transport system or other membrane channel proteins. At optimal levels, AIPs activate the two-component system (TCS), which in turn controls the expression of target genes (Sturme et al., 2007). Interspecific signal communication involves the exchange of signals between different bacterial cells. The QS system, encompassing AI-2 and the enzyme LuxS responsible for AI-2 synthesis, plays a crucial role in the intraspecific and interspecific information exchange among various bacteria. AI-2 serves as the signal molecule for sensing environmental changes and facilitating inter-bacterial communication between Gram-positive and Gram-negative bacteria (Pereira et al., 2013). It has also been proved that *luxS* gene in QS regulates various physiological activities of LAB (Gu et al., 2018).

In recent years, research on the regulation of the LuxS/AI-2 mediated QS system in LAB mainly focuses on biofilm formation. Lebeer et al. (2009) reported that the ability of mutant strains *luxS* gene knockout of *Lactobacillus rhamnosus* GG to form biofilms was decreased. Adding exogenous AI-2 precursor molecules or wild-type strains could partially restored the biofilm formation of mutant strains, but could not fully revert them to their original state. Deng et al. (2022) published similar findings after knocking out *luxS* gene of *L. rhamnosus*. On the other hand, the overexpression of the *luxS* gene in *Lactobacillus paraplantarum* L-ZS9 resulted in an increased concentration of the AI-2 signaling molecule, promoting biofilm formation. These findings demonstrated that the *luxS* gene can regulate several genes responsible for encoding transporters, membrane proteins and transcriptional regulators (Liu et al., 2018). Zhang et al. (2022) reported that the synthetic AI-2 increased the cell density of *Lactobacillus sanfranciscensis*, enhanced the bacterial cohesion, and promoted the formation of biofilms. However, the *luxS* gene deletion strain of *Lactobacillus reuteri* 100-23C formed a thicker biofilm than the wild-type strain, yet the addition of exogenous AI-2 could not fully restore the biofilm thickness to that of the wild-type strain (Tannock et al., 2005). It can be seen that LuxS/AI-2 mediated QS system plays varying roles in biofilm formation among different bacteria, including those in the LAB group. It can either promote or inhibit biofilm formation, which needs further research. In the context of the LuxS/AI-2 mediated QS system, the *pfs* gene, in addition to *luxS*, serves as a key component in the synthesis of the signaling molecule AI-2 (Han and Lu, 2009). The *pfs* gene encodes the S-adenosine homocysteine nucleotide enzyme (Pfs), responsible for the hydrolysis of S-adenosine homocysteine (SAH) into S-nucleoside homocysteine (SRH) and adenine. SRH continues to participate in the synthesis of AI-2 in the presence of LuxS protein (Zhao et al., 2018). For example, expressing the *pfs* gene in *Streptococcus suis* serotype 2 could recover AI-2 synthesis, not the *luxS* gene (Wang et al., 2013b). Consequently, it becomes evident that the key genes governing AI-2 synthesis vary among different strains.

### 2.2 Second messenger molecules involved in regulation

Cyclic di-AMP (c-di-AMP) and cyclic di-GMP (c-di-GMP) are second messengers present in the cytoplasm, with c-di-GMP being particularly involved in the regulation of bacterial biofilm formation (Hickman and Harwood, 2008). An increase in cytoplasmic c-di-GMP concentration inhibits bacterial motility, reducing it to low or no activity. This reduced motility not only facilitates cell adhesion to surfaces, but also plays a crucial role in biofilm maturation (Samrot et al., 2021). Additionally, the increase in c-di-GMP levels enhance the transcription of genes responsible for exopolysaccharide synthesis, thereby promoting the formation of biofilms (Dial et al., 2021). However, the role of c-di-GMP in the formation of *Lactobacillus* biofilm remains underexplored. He et al. (2018) confirmed the genes related to c-di-GMP metabolism in *Lactobacillus acidophilus*, including *dgcA*, *pdeA*, *pdeB*, *nrnA*, *gtsA* and *gtsB*, and also proved that these genes jointly participated in regulating the formation and co-aggregation of extracellular polymers of *Lactobacillus acidophilus* through bioinformatics and biochemical analysis tests.

### 2.3 Multiple genes involvement in macro-regulation

The regulatory mechanisms involved in each stage of LAB, from planktonic state to biofilm state, are very complex. Whether in the adhesion, growth or maturity stage, a multitude of gene expressions and the transfer of signal molecules participate in regulating biofilm formation. The key genes regulating biofilm formation are different in different strains, so it is necessary to study them from an overall perspective. Recent advancements in modern biotechnology, such as the genome, transcriptome, proteomics and metabolomics in microorganisms research has deepened understanding of the regulatory mechanisms in LAB biofilm. This is expected to lay a theoretical foundation for the targeted improvement of the production performance of LAB in the food industry. Yang et al. (2021) analyzed the key genes of AI-2 synthesis in *Leuconostoc citrem* 37 using whole genome sequencing. Five genes (*metK, DNMT/dcm, pfs, luxS* and *mmuM/BHMT2*) were involved in AI-2 synthesis, in which the *pfs* and *luxS* played an important role. In addition, the signaling pathways involved in biofilm and quorum sensing may also involve genes related to carbon metabolisms, energy metabolisms, amino acid metabolisms, signal transduction and cell membrane transport, such as *ciaH, ciaR* and 26 other genes. Sun et al. (2020) described the characteristics of the biofilm formed by *L. plantarum* J26 and clarified its metabolic pathway based on transcriptome sequencing. The findings revealed that 1,051 genes significantly differed in the planktonic and biofilm state, among which 513 genes were up-regulated, and 538 were down-regulated. These genes were closely related to pyrimidine and glycerol metabolism, amino acid synthesis, stress response, enzyme synthesis and quorum sensing. Liu et al. (2021) analyzed the metabolic characteristics of planktonic and biofilm cells of *Lactobacillus paraplantarum* L-ZS9 by untargeted metabolomics, and the results suggested a significant distinctions, with biofilm cells displaying higher activity in amino acids and carbohydrate metabolism compared to planktonic cells. This suggest that *Lactobacillus* biofilms undergo notable metabolic adaptations to support biofilm formation and stability.

### 2.4 Influence of other factors on biofilm formation

The formation of *Lactobacillus* biofilm is not only related to QS system and c-di-GMP signaling system, but also to bacterial hydrophobicity, acid-base charge, self-polymerization and environmental factors, such as pH value, temperature, carbon source, metal ion, contact surface, and water erosion. The hydrophobicity and electrostatic interaction between the cell surface or between the cell surface and the object surface played a leading role in the non-specific adhesion process of *Lactobacillus*. These interactions influence how *Lactobacillus* cells adhere to various surfaces, which is essential for biofilm formation and colonization in different environments. In particular, hydrophobicity has been used as an important index to evaluate the adhesion of *Lactobacillus*. Li et al. (2023) found that *L. plantarum* Y42 exhibits different phenotypic properties in its planktonic and biofilm forms. In particular, the biofilm form demonstrated enhanced surface properties, including increased autoaggregation ability, hydrophobicity, acid-base charge, and adhesiveness, compared to its planktonic form. Therefore, surface physicochemical modification of materials can improve the adhesion of *Lactobacillus*. For example, the hydrophilicity of a dopamine-modified polypropylene fiber membrane was improved, which in turn promoted the biofilm formation of *Lactobacillus paracasei* (Zhao et al., 2016).

The effect of pH value on the adhesion ability of *Lactobacillus* was also different among strains, and an acidic environment is more favorable to adhesion. In acidic environments, *L. acidophilus* BG2FO4 secretes a protein molecule that acts as a bridging protein, mediating the connection between bacteria and cell receptors (Coconnier et al., 1992). Additionally, the effect of temperature on both the growth and biofilm formation of *Lactobacillus* strain varies. Ramírez et al. (2015) studied the effects of temperature on seven *L. plantarum* strains and found distinct differences among them. In 5 strains, as the temperature increased, their growth decreased, but the amount of biofilm formation increased. This suggests that while higher temperatures may inhibit cell proliferation in certain *Lactobacillus* strain, they can also stimulate biofilm formation, possibly as a stress response mechanism.

The carbon source had great influence on the biofilm formation, and it was related to the utilization rate of carbon source and the preference of strain. Glucose is the carbon source commonly used for culturing *Lactobacillus*, and most *Lactobacillus* show an increase in biofilm formation as the amount of glucose increases (Ramírez et al., 2015; Akoglu, 2020). The metal ions exert a substantial influence on LAB biofilm formation, and the effects varied among strains. *Lactobacillus gasseri*, *Lactobacillus delbrueckii*, *L. reuteri* and *L. rhamnosus* isolated from the vagina did not produce biofilms in MRS without MnSO_4_ (Terraf et al., 2012). Similarly, the same result was found in the study of *L. plantarum* WCFS1 and six *L. plantarum* strains isolated from food spoilage, but *L. rhamnosus Gr18* increased the biofilm amount by 27.9% in manganese deficient environment compared to normal environment (Liu et al., 2024).

The contact surface materials commonly used in the food industry are stainless steel, glass and polystyrene. Studies had shown that *L. rhamnosus* GG had the strongest adhesion on stainless steel compared to glass, and the weakest was on polystyrene. The *luxS* gene, which is associated with the regulation of biofilm formation, was upregulated at 24 and 48 h on polystyrene and stainless steel supports, respectively (Nahle et al., 2023). In addition, the contact surface roughness has a significant influence on the adhesion of cells. The surface roughness of nano- and micro-roughness can enhance the initial adhesion of cells by increasing the contact area between the cells and the interface (Ionescu et al., 2012). Hu et al. (2019) cultivated the biofilm of *L. plantarum* on electrospun nanofiber membranes, which had excellent resistance to gastrointestinal fluids. Yogurt produced using nanofiber membranes containing *L. plantarum* biofilm as a starter culture has shown excellent fermentation properties, which shortens the fermentation time and makes the survival rate of probiotics higher during the shelf life of yogurt. According to Huijboom et al. (2024), *L. plantarum* biofilm structure and matrix, physiological state and stress resistance of cells is strain dependent and strongly affected under flow conditions.

Some non-signaling system-related chemicals can also alter *Lactobacillus* biofilms formation. The resveratrol can change the physicochemical properties of the surface of *Lactobacillus paracasei*, thereby enhancing the aggregation of cells and promoting adhesion and biofilm formation (Al Azzaz et al., 2020). The serotonin can promote the transport of quorum sensing signaling peptides in *Enterococcus faecium*, increase the abundance of some proteins related adhesion, and enhance the ability of cell self-aggregation and biofilm formation (Scardaci et al., 2021). The organic selenium regulated QS system of *L. paracasei* by binding two crucial receptor proteins (histidine protein kinase HPK and periplasmic binding protein LuxP) from specific sites, and promoted the biofilm formation (Lou et al., 2024). Therefore, *Lactobacillus* biofilm formation can be regulated by exogenous addition of these chemicals.

## 3 Relationship between *Lactobacillus* biofilm and adhesion

The adhesion of *Lactobacillus* to the surface is the first step for *Lactobacillus* to play a probiotic role. At the early stage of adhesion, whether or not the bacteria can attach to the cell surface is determined by the physicochemical properties of the bacterial surface, mainly by the properties of surface proteins. Bacterial initial adhesion and biofilm formation are controlled by different genes, and bacterial initial adhesion is an important prerequisite for biofilm formation (van Merode et al., 2006). For instance, the addition of mucin to MRS medium without glucose increased the biofilm formation ability of *L. rhamnosus* by 20% (Lebeer et al., 2007). At the later stage of adhesion, more extracellular matrix is secreted to the outside of the cell with the continuous proliferation of bacteria, so that the bacteria accumulate in large numbers, and finally form biofilm. The formation of *Lactobacillus* biofilm can greatly improve the adhesion ability of the bacteria. The extracellular matrix plays a key role in the later stage, especially exopolysaccharides. It had been reported in comparison to the wild-type *L. rhamnosus*, the mutant strain containing genes related to extracellular polysaccharide synthesis exhibited stronger biofilm formation ability (Lebeer et al., 2009).

The adhesion ability of *Lactobacillus* is related to its surface polysaccharides and membrane proteins. It had been found that *L. plantarum* WCFS1 had a similar QS system lamBDCA to *Staphylococcus aureus*. The adhesion of mutant *lamA* to glass surface is lower than that of wild-type strains, which is the first time that non-pathogenic agr systems have been found to encode self-inducible peptides and participate in the regulation of adhesion (Sturme et al., 2005). Because biofilm formation is closely related to the QS system mediated by the signaling molecule AI-2, it has been found that the signaling molecules of the QS system can influence the adhesion of LAB. The AI-2 signaling molecules produced by *L. acidophilus* NCFM can enhance the formation of biofilms and ultimately increase the adhesion to intestinal epithelial cells (Buck et al., 2009). Zhang et al. (2022) compared the effects of endogenous and synthetic AI-2 on the growth of *L. sanfranciscensis*. The synthetic AI-2 increased the cell density and cohesive force of *L. sanfranciscensis*. The adherence of *luxS* mutant *L. acidophilus* to Caco-2 decreased by 58% compared with the wild type (Buck et al., 2009). The ability of *L. plantarum* KLDS1.0391 to adhere to Caco-2 cells was drastically decreased by the *luxS* gene knockout, speculating that luxS/AI-2 mediated QS may affect the adhesion of LAB and regulate biofilm (Jia et al., 2018). Therefore, it is of great significance to utilize QS system to regulate the formation of *Lactobacillus* biofilms, thereby improving the adhesion ability of *Lactobacillus* and enhancing the probiotic effect of *Lactobacillus*.

## 4 Stress resistance of *Lactobacillus* biofilm

Biofilm is a self-protective mechanism formed by microorganisms in an adverse environment. They exhibit a natural capacity for self-production of extracellular polymeric substances (EPS), which include exopolysaccharides, proteins, and extracellular DNA. These substances enhance their adhesion to surfaces, forming a thick protective barrier. In addition to providing a structural barrier, *Lactobacillus* also tend to survive in the form of biofilm when faced with adverse environmental stress, thus enhancing the stress resistance of the bacteria. The stress resistance of *Lactobacillus* biofilm to different environments is shown in Table 1. The protective effect of biofilm is thought to be mainly due to the fact that the extracellular polymers of biofilm shield the damage of toxic compounds, antibiotics and enzymes to the embedded bacteria, and the diffusion of harmful substances can be hindered by the extracellular polymers (Nguyen et al., 2022). Therefore, strains located deeper in the biofilm can be better protected. In addition, *L. plantarum* biofilms have been shown to withstand harsh conditions in fermented foods due to their robust EPS matrix, which protects against desiccation and nutrient depletion (Wang et al., 2021).

**TABLE 1:** Types of stress that *Lactobacillus* biofilms are able to withstand.

Lactobacillus species	Stress agents	References
*Lactobacillus plantarum*, *Lactobacillus brevis, Lactobacillus fructivorans*	Acetic acid, ethyl alcohol	Kubota et al., 2008
*Lactobacillus plantarum subsp plantarum* JCM 1149	Organic acid (acetic acid, citric acid, lactic acid, malic acid), ethyl alcohol, sodium hypochlorite	Kubota et al., 2009
*Lactobacillus rhamnosus* PTCC 1637, *Lactobacillus plantarum* PTCC 1745	Enrofloxacin, sulfadiazine, tetracycline, terramycin	Rezaei et al., 2022
*Lactobacillus plantarum*	Acid, alkali, bile salt, artificial gastric juice, artificial intestinal juice	Zhang et al., 2021
*Lactobacillus plantarum*	Acid, ethyl alcohol	Pannella et al., 2020

Recent research shows that when exposed to acidic environment, *Lactobacillus* activate a complex stress response that leads to significant physiological adaptations. This stress response often involves upregulation of acid tolerance mechanisms, including the F0F1-ATPase system and the production of chaperones that stabilize cellular proteins on low pH conditions (Zhang et al., 2022; Wang et al., 2021). This adaptation not only enhances survival but also improves intercellular communication and resource sharing through quorum sensing, particularly via the AI-2 signaling pathway (Papadimitriou et al., 2016). Furthermore, the physiological changes triggered by acidic stress responses in *Lactobacillus*, such as modifications in the cell surface properties and membrane liquid composition have been shown to provide cross-protection against additional stresses, like oxidative and osmotic pressures (Papadimitriou et al., 2016).

As an important regulatory mechanism involved in bacterial metabolism, it is of utmost importance to study the stress mechanism of LAB under different environmental stresses from the LuxS/AI-2 mediated QS system. At present, some progress has been made in this aspect. A total of 72 genes showed differential expression in *L. reuteri* after being treated in the acidic environment, among which the expression level was *luxS* gene up-regulated by approximately 3–4 times (Wall et al., 2007). The *luxS* gene expression was significantly up-regulated in all three strains of *L. plantarum* F, *Lactobacillus sakei* L4 and *L. plantarum* R, in which transcription level was positively associated with salt concentration under high nitrate stress, but differentially among the three strains (Lin et al., 2015). Transcription level of the *luxS* gene also appear to be upregulated under nutrient deficient conditions (Gu et al., 2018). Given that the *luxS* gene regulates the synthesis of the signaling molecule AI-2, the influence of AI-2 on the stress resistance of LAB exhibits a certain strain specificity and concentration dependence. Adding the exogenous signaling molecule AI-2 can improve the bile salt tolerance of *L. sanfranciscensis* (Zhang et al., 2022). The exogenous addition of AI-2 by Gu et al. (2021) significantly improved the tolerance of *L. plantarum* to bile salts but did not improve the tolerance to acid. Overexpression of *luxS* gene promoted AI-2 synthesis and enhanced the heat tolerance of *L. paraplantarum* L-ZS9 (Liu et al., 2018). Meanwhile, the deletion of *luxS* gene will have some effect on the stress resistance and normal physiological function of LAB. Bove et al. (2012) knocked out the *luxS* gene of *L. plantarum* KLDS1.0391 and showed no significant difference in the number of viable cells compared to the wild-type strain under normal culture conditions, but the acid and bile salt tolerance of the strain with the deletion of *luxS* gene was significantly reduced under high acid and high bile salt environments.

In addition to the *luxS* gene, other genes related to the QS system were also associated with the stress resistance of strains, such as the gene *ftsH* encoding FtsH protein in the AAA (ATPase associated with different cellular activities) family, a ubiquitous membrane-bound, ATP-dependent metalloproteinase. Some studies have shown that the mutation of *ftsH* caused the loss of FtsH protease in *L. plantarum* WCFS1, which resulted in poor heat and salt tolerance of the mutant and reduced the ability to form biofilm. On the contrary, the overexpression of FtsH enhanced the heat tolerance, salt tolerance and biofilm formation ability of *L. plantarum* (Del Pozo, 2018). In the *L. sanfranciscensis*, the addition of artificial AI-2, can up-regulate the expression of *ftsH* gene, promoting biofilm formation and improving the bile salt tolerance (Zhang et al., 2022). In addition, in the mixed-species biofilms formed by *Streptococcus thermophilus* with *Lactobacillus bulgaricus*, *Lactobacillus helveticus*, *Lactobacillus equinosus* and *L. paracasei*, have been shown to offer better microbial protection due to the increased biofilm mass (Yao et al., 2022).

A large number of studies have proved that *Lactobacillus* biofilms exhibit strong tolerance to the environment. Studies on *Lactobacillus* stress responses have been relatively comprehensive (Baig et al., 2022), especially regarding acid resistance mechanism (Wang X. et al., 2022). However, studies specifically examining the link between stress responses and biofilm formation in *Lactobacillus* remains limited. Therefore, future research on stress resistance of *Lactobacillus* biofilms should focus on exploring the specific stress responses induced by various environmental factors and elucidating the mechanisms behind biofilms’ enhanced resistance. This mechanistic insight will support strategies to improve the resilience of *Lactobacillus* through biofilm-based approaches.

## 5 Application of *Lactobacillus* biofilms in food industry

Historically, *Lactobacillus* has been mostly used in food industry in the form of planktonic or cell-free metabolites, such as food starter culture and oral bacterial solution. Although most *Lactobacillus* can play a probiotic role in food, the strain will form a biofilm due to changes of environment, which leads to food spoilage. It has been reported that many *Lactobacillus* can form biofilms that can affect the quality of meat, cheese, sake, beer and salads. For example, *Lactobacillus fructivorans* can cause mayonnaise and miso to deteriorate. *Lactobacillus acetotolerans* and *Lactobacillus brevis* can cause the deterioration of vinegar, and *L. plantarum subsp. plantarum* may cause the contamination of common food production plants as well as spoilage of pickles (Somers et al., 2001; Kubota et al., 2009). However, *Lactobacillus* biofilms also exhibit unique features in terms of stress resistance and antibacterial, which have been used for food production and human health. The application of *Lactobacillus* biofilm in food mainly involves three aspects, prevent adhesion of pathogens, control spoilage organisms and improve food safety (Jara et al., 2020). Studies have shown that *Lactobacillus* biofilm can control the growth of *Listeria monocytogenes*, *Salmonella* Typhimurium and *Escherichia coli* O157:H7 (Silva et al., 2020). In addition, pathogen inhibition analysis of *Escherichia coli*, *Staphylococcus aureus*, and *L. monocytogenes* suggested a significant distinction between the planktonic and biofilm of *L. reuteri* DSM 17938 (Hu et al., 2022a). In addition to inhibiting food spoilage bacteria, it has also been found to remove common toxins in foods. *L. rhamnosus* biofilm was found to be able to remove aflatoxin M1 from milk by binding it to its cell surface components, reducing the toxin’s bioavailability and thereby potentially enhancing the safety of dairy products (Assaf et al., 2019). The tremendous benefits of *Lactobacillus* biofilms have not only been noticed against bacterial pathogens but also against fungi, especially in clinical environments. Therefore, the antifungal activities of *Lactobacillus* biofilms are also important in order to expand the therapeutical approaches to fungi diseases.

Biofilms formed by probiotics are considered an effective strategy against biofilms of pathogenic bacteria in certain disease intervention. They can compete with pathogenic bacteria for nutrients and space through different mechanisms of action, such as producing bacteriostatic substances, playing an immunomodulatory role, and competing with pathogenic bacteria for adhesion sites. Many researchers are trying to use probiotics as an alternative to control infection and introduce promising treatment alternative to infections (Guerrieri et al., 2009). Table 2 shows some examples of lactic acid bacteria biofilm inhibiting foodborne or pathogenic microorganisms.

**TABLE 2:** Examples of pathogens inhibited by *Lactobacillus* biofilms.

*Lactobacillus* species	Pathogenic microbial species	Details	References
*Lactobacillus plantarum*	*Listeria monocytogenes*	Effective in reducing the amount of planktonic or adherent *Listeria monocytogenes* and more effective under acidic conditions	Song et al., 2019
*Lactobacillus rhamnosus*	*Escherichia coli*	Effective in inhibiting the formation of *Escherichia coli* biofilm and destroying the mature biofilm of *Escherichia coli*	Merino et al., 2019
*Lactobacillus sakei, Lactobacillus curvatus*	*Listeria monocytogenes, Salmonella* Typhimurium, *Escherichia coil O157:H7*	*Lactobacillus* are able to form protective biofilms and prevent biofilm formation by *Listeria monocytogenes*, *Salmonella* Typhimurium, *Escherichia coli*	Gómez et al., 2016
*Lactobacillus plantarum*	*Salmonella* Enteritidis, *Bacillus cereus, Pseudomonas fluorescens, Aeromonas hydrophila*	*Lactobacillus plantarum* AP21 biofilm can inhibit the growth of these microorganisms	Wallis et al., 2019
*Lactobacillus rhamnosus, Lactobacillus plantarum*	*Staphylococcus*	*Lactobacillus reuteri* ATCC 7469 and *Lactobacillus plantarum* 2/37 could form a biofilm to replace staphylococcus biofilm causing cow mastitis.	Al-Hadidi et al., 2021
*Lactobacillus reuteri*	*Clostridium perfringens*	*Lactobacillus reuteri* biofilm reduced the incidence of necrotizing enterocolitis, severity, and neurocognitive sequelae	Zhang et al., 2020
*Lactobacillus plantarum*	*Streptococcus mutans*	*Lactobacillus plantarum* FB-T9 could prevent dental caries, significantly reducing the level of *Streptococcus mutans* in the tooth surface of rats	Scatassa et al., 2015
16 vaginal *Lactobacillus strains* (belonging to *Lactobacillus crispatus, Lactobacillus gasseri, Lactobacillus vaginalis, and Lactobacillus plantarum species)*	*Candida* clinical isolates, such as *Candida albicans, Candida glabrata, Candida lusitaniae, Candida tropicalis, Candida krusei, and Candida parapsilosis*	The biofilm cell-free supernatant from *L. crispatus* and *L. plantarum* strains exerted the strongest fungistatic activity against all candida isolates compared to planktonic cell-free supernatant.	Parolin et al., 2021
*Lactobacillus rhamnosus*	*Aspergillus flavus*	Reducing the growth of toxin-producing *Aspergillus flavus* by producing antifungal compounds in food systems	Magnusson et al., 2003

The use of *Lactobacillus* biofilms can extend the shelf life of microbial starters, reduce fermentation times, and enhance the characteristic flavors of fermented food. Hu et al. (2019) cultivated the biofilm of *L. plantarum* on electrospun nanofiber membranes, which had excellent resistance to gastrointestinal fluids. Yogurt produced using nanofiber membranes containing *L. plantarum* biofilm as a starter culture has shown excellent fermentation properties, which shortens the fermentation time and makes the survival rate of probiotics higher during the shelf life of yogurt. In the production of traditional cheeses, Italian Ragusano cheese and French Salers cheese are made from raw milk, while oak barrels used for fermentation and maturation are rich in microbial biofilms, which are mainly composed of LAB and have an important influence on the unique flavor of cheese (Carpino et al., 2017; Furukawa, 2015).

*Lactobacillus* biofilm is used to produce metabolites, which is conducive to improving the production efficiency. Bastarrachea et al. (2022) use the biofilm formed by *L. paracasei* on the chitosan-modified polypropylene material to ferment lactic acid. The findings revealed that the production rate of lactic acid has been significantly improved. The fermentation system has the characteristics of high yield, good continuity and stability, which can maintain a high yield for a long time. Similarly, Zhao et al. (2016) found that a dopamine-modified polypropylene fiber membrane can promote the biofilm formation of *L. paracasei*, and the biofilm had excellent stress resistance. The purity of L-lactic acid produced by fermentation in the biofilm reactor was maintained at more than 99%. In contrast, the reactor had no fermentation delay period, effectively improving biological fermentation efficiency.

The fermentation process often involves multiple microbial species including *Lactobacillus*, yeast, and acetic acid bacteria. These microbial communities always exist and play a role in the form of mixed-species biofilm. The application of biofilms formed by *Lactobacillus* and other species of microorganisms in fermented food production are shown in Table 3. A better understanding of the formation, spatial distribution, systematic succession and function of multi-species biofilms can help researchers better control and utilize biofilms during fermentation and help improve the yield, quality, and safety of fermented products.

**TABLE 3:** Application of multispecies biofilms in food productions.

Fermented products	Main microorganisms	The role of biofilms	References
Acetic acid beverage	*Lactobacillus plantarum, acetic acid bacteria*, *yeast*	Ethanol and acetic acid were produced efficiently, and the tolerance of strains to ethanol and acid was improved	Furukawa, 2015
Fukuyama pot vinegar	*Lactobacillus plantarum*, *Saccharomyces cerevisiae*, *Acetobacter pasteurianus*	Lactic acid produced by *Lactobacillus. plantarum* could induce *Acetobacter pasteurianus* to form biofilms on the surface of fermentation broth. *Acetobacter pasteurianus c*onverted ethanol into acetic acid	Cosetta et al., 2020
Daqu	*Lactobacillus plantarum, Lactobacillus brevis*, *Saccharomyces cerevisiae*	The mixed biofilm with *Saccharomyces cerevisiae* improved the environmental adaptability of *Lactobacillus*, and was conducive to the production of ethanol and flavor compounds by yeast and *Lactobacillus*, respectively	Fan et al., 2020
Kefir	*Acetobacter* spp., *Lactobacillus* spp., *Lactococcus* spp., *Leuconostoc* spp., *Kluyveromyces* spp.	The formation of mixed biofilm played an important role in the formation of kefir particles and protected the kefir-producing strains against acetic acid and stresses	Wang X. M. et al., 2022
Cheese	*Lactobacillus* spp., *Lactococcus* spp., *Leuconostoc* spp., *Enterococcus* spp.	The multispecies biofilms promote the formation of special flavor of cheese	Carpino et al., 2017
Kombucha	*Lactobacillus* spp*., Acetobacter* spp., *Gluconacetobacter* spp., yeast	Biofilms provided a resource storage function and inhibited the diffusion of antibiotics and invasion of exogenous microorganisms	May et al., 2019
Olive	*Lactobacillus plantarum*, yeast	The adhesion of undesirable planktonic microorganisms was inhibited during storage	Faten et al., 2016
Pickled radish	*Lactobacillus* spp., *Lactococcus* spp., *Leuconostoc* spp., yeast, *Aspergillus*	The potential pathogenic bacteria *Reuteria* and putrefying bacteria *Pseudomonas* were inhibited	Li and Liu, 2022

## 6 *Lactobacillus* biofilm encapsulation

To have any biological effect, a daily dose of probiotics of about 10^8^–10^9^ colony-forming units (CFU) is required prior and during passage through the gastrointestinal tract (Liu et al., 2019). Strategies for preserving the viability of probiotic strains have changed over time, leading to the development of the fourth-generation probiotics. Fourth-generation probiotics are represented by encapsulated probiotics, whereby bacterial cells exist in the form of biofilms. Encapsulation of LAB in the form of a biofilm involves embedding the bacteria within a protective matrix of edible colloidal substances like alginate, chitosan, or gelatin. *Lactobacillus* biofilm encapsulation protects LAB from the external environment and achieves release at the target site under conditions of active metabolism (Salas-Jara et al., 2016). Previous studies have verified that *Lactobacillu*s can be encapsulated and form biofilms within capsules. These encapsulated biofilms show improved resistance to heat, acid, and storage environments. Cheow and Hadinoto (2013) encapsulated *L. rhamnosus* biofilm with a double-layer coating, which found stronger resistance than the planktonic strains encapsulated with a double-layer coating. Heumann et al. (2020) successfully prepared calcium pectin beads in which encapsulated *Lactobacillus paracasei* ATCC334 cells showed higher resistance to acid stress, freezing-drying stress, and combined stress. He et al. (2021) encapsulated LAB with low-methoxyl pectin, stimulated the biofilm formation of encapsulated lactobacilli upon *in situ* cultivation on microcapsules. The findings revealed that the microcapsules formed by biofilm-forming bacteria are more resistant to thermal shock, freeze-drying, gastrointestinal digestion, and drugs than those formed by planktonic bacteria. Vega-Sagardía et al. (2018) encapsulated *Lactobacillus fermentum* UCO-979C with alginate, xanthan gum, and vegetable oil, and allowed the strain to form biofilm within the microcapsules, and subjected to pH 3.0 maintained the anti-*H. pylori* inhibitory activity.

Probiotic encapsulation mainly depends on the choice of wall materials, such as emulsion, gel, nanocoating, and liposomes. Each encapsulating material has its advantages and disadvantages with respect to chemical, biological, mechanical, and physical properties (Razavi et al., 2021). Generally, the wall material of microcapsules should not affect the growth and metabolism of probiotics or human health. In addition, it should also have the advantages of being widely available, affordable, edible, and able to ensure targeted release in the colon while simultaneously resisting various adverse environments. Electrospun nanofibrous scaffolds have demonstrated excellent properties in facilitating the biofilm formation of probiotics (Hu et al., 2022b; Hu et al., 2019). However, the output of electrospun nanofibrous scaffolds is limited by the existing production method. Recent studies have explored using natural materials such as polysaccharides and proteins as encapsulation materials (Table 4). Seeking green, inexpensive, and sustainable encapsulation materials that support biofilm formation is important for the successful cultivation and large-scale production of biofilm-based probiotics.

**TABLE 4:** Capsule materials and characteristics of *Lactobacillus* biofilm encapsulation.

Biofilm-like strain	Encapsulating materials	Characteristics	Reference
*Lactobacillus rhamnosus* GG (LGG)	Bacterial cellulose–pullulan	Improve the tolerance of LGG to acid and bile salt, and LGG could be successfully released in the colon	Savitskaya et al., 2024
*Lactiplantibacillus plantarum* GDMCC 1.140, *Lactobacillus rhamnosus*	Rhamnogalacturonan I-rich pectin	Improve the tolerance of strains to heat stress, H2O2 stress, osmotic pressure stress, freeze-drying stress, and the activity in the gastrointestinal tract	Chen et al., 2024
*Pediococcus pentosaceus*	Alginate	The survival ability of the strain was improved in gastrointestinal environment, refrigerator storage and acidic environment, and showed high thermal stability.	Mgomi et al., 2024
*Lactiplantibacillus paraplantarum* L-ZS9	Pectin	Improve the tolerance of the strain to acid, gastrointestinal environment and freeze drying	Liu et al., 2023
*Lactobacillus reuteri*	Porous zein/cellulose	Improve the tolerance of the strain to gastrointestinal environment, and showed a good regulatory effect on intestinal microbiota and short-chain fatty acids.	He et al., 2024
*Lacticaseibacillus rhamnosus*, *Lactiplantibacillus plantarum*	Milk	Improve the survival of these bacteria in probiotic yogurt during processing, storage, and gastrointestinal conditions	Rezaei et al., 2023

## 7 Conclusion

In conclusion, research on the *Lactobacillus* biofilms has grown significantly in recent years, highlighting their beneficial properties, such as promoting bacterial adhesion, enhancing stress resistance and preventing colonization by pathogens. Despite this progress, substantial doubts remain to be resolved. Existing studies of LAB biofilms have focused mainly on strains of the genus *Lactobacillus*, whereas reports on biofilm in strains of the genera *Bifidobacterium*, *Streptococcus*, *Leuconostoc*, and *Pediococcus* are rare. Species-specific biofilm formation mechanisms and biofilm function should be investigated. Meanwhile, the applications of *Lactobacillus* biofilm is limited by the low cell viability of planktonic cells, the detachment of surface cells, the cell release after biofilm maturation, the incomplete regulatory mechanism and the limited material diffusion within biofilms. Another issue is determining the optimal encapsulation materials and parameters for developing fourth-generation probiotics. Survival of fourth-generation probiotics has been assessed only *in vitro* and thus requires further studies. These limitations also present opportunities for advancement. The rapid development of emerging technologies, such as multi-omics, gene-editing systems, electrostatic spinning and electrostatic spraying, has provided more options for studying the *Lactobacillus* biofilms. Continuous increases in the understanding of the *Lactobacillus* biofilm will undoubtedly help to enhance resistance to stress, inhibit harmful microorganisms, and enable more remarkable breakthroughs in improving product quality and flavor, and producing high-value products.
